# Serum miRNA Signature in Moyamoya Disease

**DOI:** 10.1371/journal.pone.0102382

**Published:** 2014-08-05

**Authors:** Dongwei Dai, Qiong Lu, Qinghai Huang, Pengfei Yang, Bo Hong, Yi Xu, Wenyuan Zhao, Jianmin Liu, Qiang Li

**Affiliations:** 1 Department of Neurosurgery, Changhai Hospital, Second Military Medical University, Shanghai, China; 2 Department of Laboratory Medicine, Changhai Hospital, Second Military Medical University, Shanghai, China; Sanjay Gandhi Medical Institute, India

## Abstract

Moyamoya disease (MMD) is a cerebrovascular disease characterized by progressive stenosis of the intracranial internal carotid arteries and their proximal branches. However, the etiology of this rare disease remains unknown. Serum microRNA (miRNA) profiles have been screened to identify novel biomarkers of prognostic values. Here, we identified serum miRNAs that might play an important role in the pathogenesis of MMD. A genome-wide miRNA array analysis of two pooled serum samples from patients with MMD and controls revealed 94 differentially expressed serum miRNAs, including 50 upregulated and 44 downregulated miRNAs. In an independent MMD cohort, real-time PCR confirmed that miR-106b, miR-130a and miR-126 were significantly upregulated while miR-125a-3p was significantly downregulated in serum. GO analysis showed that the differentially expressed serum miRNAs were enriched in metabolic processes, transcription and signal transduction. Pathway analysis showed that the most enriched pathway was mTOR signaling pathway with 16 potential, functional targets. Finally, we found that 16 and 13 aberrant serum miRNAs coordinately inhibited RNF213 and BRCC3 protein expression at the posttranscriptional level, respectively, resulting in defective angiogenesis and MMD pathogenesis. To our knowledge, this is the first study to identify a serum miRNA signature in MMD. Modulation of the mechanism underlying the role of serum miRNAs in MMD is a potential therapeutic strategy and warrants further investigations.

## Introduction

Moyamoya disease (MMD) is an idiopathic disorder manifesting stenosis or occlusion of a terminal portion of the internal carotid artery (ICA) or a proximal portion of the anterior cerebral arteries and the middle cerebral arteries (ACAs, MCAs) as well as abnormal vascular networks near the occlusive or stenotic lesions, as shown by cerebral angiography [1].

Histopathology of the carotid arteries reveals fibrocellular thickening of the intima. Moyamoya vessels show dilated perforating arteries with fibrin deposits, fragmented elastic laminae, and microaneurysms [2], [ 3].

Radiological findings such as computed tomography (CT) perfusion [4] and magnetic resonance imaging (MRI), are necessary for accurate diagnosis, especially MRI, which assists easier detections of asymptomatic patients with familial MMD [2]. In clinical diagnosis and treatment of cerebrovascular diseases, dynamic susceptibility contrast magnetic resonance has been widely used [5]. The “ivy sign” refers to the diffuse leptomeningeal enhancement that is found on post contrast MR images in patients with MMD or moyamoya syndrome [6]. Due to slow flow, prominent leptomeningeal collaterals result in vivid contrast enhancement and high signal on Fluid-Attenuated Inversion Recovery (FLAIR) [7]. The appearance is a reminiscence of the brain covered with ivy. High-resolution magnetic resonance imaging (HR-MRI) manifested smaller, concentric occlusive lesions, which are rarely enhanced in comparison with symptomatic intracranial atherosclerotic disease (ICAD) [8]. Arterial spin labeling (ASL), a completely noninvasive modality, is employed to investigate details of cerebral blood flow [9].The incidence of MMD worldwide in individuals with diverse ethnic backgrounds, including American and European populations is well established. However, the disease is extremely uncommon in non-Asian populations [10], [ 11]. The etiology of MMD is still unknown [12]. It is, therefore, essential to investigate the mechanisms underlying the development and progression of the disease.

Genetic linkage analyses unraveled five candidate loci for MMD including: chromosome 3p24–26, 6q25, 8q23, 12p12 and 17q25 [13], [ 14]. Genome-wide association studies (GWAS) also revealed several susceptibility genes: ACTA2, RPTOR, PDGFRB and TGFB1 [15], [ 16], [ 17]. RNF213 was identified as an MMD susceptibility gene in a genome-wide, locus-specific association study. It has since been confirmed in a recent large scale sequencing analysis [18], [ 19], [ 20]. In the near future, the pathogenesis of MMD might be determined by genetic analyses. Identification of the relevant genes may be very promising for the development of novel gene therapies and prevent the occurrence of MMD [2], [ 21], [ 22].

MicroRNAs (miRNAs) are short non-coding RNAs that regulate gene expression by binding to the 3′-untranslated regions (3′-UTRs) of specific mRNAs. MiRNA expression signatures have prognostic values [23], [ 24], [ 25], [ 26], [ 27]. Recently, novel biomarkers for disease diagnosis and prognosis have been identified in serum miRNA [28], [ 29], [30]. For example, miRNA-21, miRNA-155, miRNA-196a and miRNA-210, were found to be elevated in the plasma of patients with pancreatic carcinoma [31], [ 32]. Serum miRNAs are therefore, potential, independent prognostic factors compared with biomarkers derived from target tissues.

In this study, we hypothesized that serum miRNAs are candidate biomarkers in MMD. We systematically screened serum miRNAs by using miRNA arrays and validated the results by miRNA real-time PCR. Bioinformatics analyses revealed several important pathways and serum miRNAs potentially involved in the disease. To our knowledge, this is the first study to identify serum miRNA signature in MMD.

## Methods

### Sample preparation and RNA extraction

Written informed consents on the use of samples for analysis were obtained from all participants and/or their guardians before entry. The study was approved by the Ethics Committee Review Board of Changhai Hospital at Shanghai where the study was carried out. We included 10 adults with MMD diagnosed by digital subtraction angiography (DSA) along with 10 adults serving as controls. The diagnostic criteria for MMD were based on the guidelines published in 1997 by the Research Committee on the Spontaneous Occlusion of the Circle of Willis of the Ministry of Health and Welfare, Japan [2]. The inclusion criteria were: bilateral angiographic identification of severe stenosis or occlusion of the distal internal carotid, proximal middle cerebral, and anterior cerebral arteries, associated with an abnormal network of collateral vessels [33]. The exclusion criteria involved the presence of secondary moyamoya phenomenon caused by atherosclerosis, meningitis, Down syndrome, hyperthyroidism, neurofibromatosis, leptospiral infection, or prior skull-base radiation therapy. The regional distribution of MMD is indicated in Table 1. The control group of healthy adults was of a similar age and sex ratio as the adult patients with MMD. All patients and healthy subjects were of an ethnically homogeneous Han Chinese origin.

**Table 1:** Clinical characteristics of 20 MMD patients.

No	Presentation	Initial CT or MR Image Findings	Suzuki Stage	Region of Origin(Province)
***Blood samples of MMD patients used in the microarray analysis***				
1	IVH	intracerebral hematoma(rt periventricular region)	rt5 lt4	Shanghai
2	Infarction	infarction (lt frontal white matter)	rt3 lt2	Beijing
3	TIA	lacunae	rt3 lt3	Guangdong
4	TIA	normal	rt3 lt2	Anhui
5	TIA	normal	rt2 lt3	Henan
6	TIA	lacunae	rt3 lt4	Shandong
7	Infarction	infarction (rt temperal white matter)	rt4 lt4	Shanghai
8	TIA	normal	rt3 lt2	Hubei
9	IVH	subarachnoid hemorrhage	rt4 lt3	Jiangsu
10	TIA	lacunae	rt1 lt2	Sichuan
***Blood samples of MMD patients used in the Real-Time PCR analysis***				
1	TIA	lacunae	rt2 lt3	Hubei
2	TIA	normal	rt3 lt2	Xinjiang
3	TIA	lacunae	rt3 lt3	Anhui
4	Infarction	infarction (rt occipital white matter)	rt5 lt4	Zhejiang
5	IVH	intraventricular hemorrhage(rt)	rt4 lt4	Shandong
6	TIA	lacunae	rt3 lt3	Jiangsu
7	TIA	normal	rt2 lt3	Qinghai
8	TIA	normal	rt3 lt2	Fujian
9	Infarction	infarction (lt temporal white matter)	rt4 lt4	Shanxi
10	IVH	intracerebral hematoma(lt periventricular region)	rt4 lt4	Shanghai

CT  =  computerized tomography; IVH  =  intraventricular hemorrhage; MR  =  magnetic resonance; TIA  =  transient ischemic attack; lt  = left; rt  =  right.

We collected 5 ml of venous blood from each participant using a procoagulant drying tube. The whole blood was centrifuged at 1500 g for 10 min at room temperature, and at 13800 g for 15 min at 4°C to completely remove cell debris. To detect the general signatures of serum miRNAs for MMD, we pooled serum samples from 10 patients and 10 controls respectively. Trizol Reagent (Invitrogen, Carlsbad, CA) was used for serum denaturation. Qiagen miRNeasy Mini kit (Qiagen, Valencia, CA) was used for RNA collection and purification, according to the manufacturer's protocol. We collected independent serum samples, including another 10 MMD patients and 10 controls, for miRNA validation by real-time PCR. Each new serum sample was separately used for RNA extraction and real-time PCR.

### MiRNA microarray

We conducted three independent experiments using an Agilent Human 8×60 K miRNA array with the two pooled samples. The integrity of the total RNA was checked using an Agilent 2100 Bioanalyzer (Agilent, Santa Clara, USA). We synthesized the cDNA and biotinylated cRNA and hybridized to the array. Data were acquired using the Agilent G2565BA Microarray Scanner System and Agilent Feature Extraction Software. Probe intensities were normalized using Percentile Shift implemented in GeneSpring 12. Accession number(GSE45737)was obtained after submitting the dataset to Gene Expression Omnibus. Differentially expressed miRNAs were identified through fold- change filtering (fold change>2).

### Real-time PCR

Additional serum samples were collected independently from 10 adult patients with MMD and 10 adult controls for miRNA validation. Each individual serum sample was separately used for RNA extraction and real-time PCR. The inclusion and exclusion criteria of MMD patients used in RT-PCR were identical to the RNA extraction protocol. The regional distribution was indicated in Table 1. All MMD patients and controls were of an ethnically homogeneous Han Chinese origin.

The miRNAs were quantified by using real-time PCR based on SYBR Green. The total serum RNAs were isolated using TRI Reagent BD (MRC, TR126), based on the manufacturer's modified instructions. In brief, 100 µl of plasma was added to 0.75 ml of TRI Reagent BD supplemented with 20 µl of acetic acid (5 mol/L). We used 5 µl of synthetic *C.elegans* miRNA (cel-miR-39, 50 pmol/L) as control [34], [ 35], [ 36] before chloroform extraction. We then precipitated RNA using isopropanol at −20°C, and resuspended the pellet in RNase-free water. In the absence of a unified internal reference for serum miRNA detection, we used the exogenous synthetic cel-miR-39 as control [37].The miRNeasy Serum Kit(Qiagen)was used to detect serum miRNA level [36]. First, we synthesized cDNA using small RNA from serum samples using miScript II RT Kit (Qiagen), according to the manufacturer's instructions. Real-time PCR was performed on a Rotor-Gene 3000A PCR Machine. The forward primers used in the PCR amplification reaction were as follows (5′ to 3′ shown):

GCCCTAAAGTGCTGACAGTGCAGAT for miR-106b; CCTACCACAGGGTAGAACCACGG for miR-140-3p; CAGCAGTGCAATGTTAAAAGGGCAT for miR-130a; GCCTCGTACCGTGAGTAATAATGCG for miR-126; and ACAGGTGAGGTTCTTGGGAGCC for miR-125a-3p. The universal reverse primers were provided by the miScript SYBR Green PCR Kit (Qiagen).

### Bioinformatics analysis

In this study, we used TargetScan (http://www.targetscan.org/, TargetScanHuman 6.2) to identify the targets of differentially expressed miRNAs. In TargetScan, biological targets of miRNAs are predicted by searching for the presence of conserved 8 mer and 7 mer sites that match the seed region of each miRNA. We defined the predicted target genes by no less than 12 differentially expressed miRNAs as potential functional targets in MMD.

To determine the biological relationship between the potential miRNA target genes, Gene Ontology [37] and KEGG [38], [ 39] pathway enrichment analysis was performed using DAVID (http://david.abcc.ncifcrf.gov/)[40]. The significance threshold was set to 0.05 in our enrichment analysis.

### Statistical Analysis

We used SPSS 11.0 (SPSS, Chicago, IL) to analyze the dataset from miRNA microarray experiments. All data were represented by mean ± SD. Statistical significance was determined at P<0.05. In order to screen the aberrant serum miRNAs in MMD, the criterion (more than 2-fold) was set. We detected the plasma miRNA levels both in healthy control and Moyamoya patients by real-time PCR. The data were evaluated by Shapiro-Wilk test to see whether they followed the normal distribution. The basis to declare a certain parameter as normally distributed was adjusted to P = 0.20. For the data that did not fit the normal distribution, Kruskal-Wallis test was performed. For the data of normal distribution, Levene's test of homogeneity of variance was further performed. When the data fitted the homogeneity of variance, One-way ANOVA was applied; and for the data that did not fit the homogeneity of variance, Kruskal-Wallis test was performed.

## Results

### Serum miRNAs were aberrantly expressed in MMD

Baseline clinical demographics were summarized in Table 1 and Table 2. The cases and controls were well matched in age, sex, routine blood tests, liver function and lipid screening. Total cholesterol and low density lipoprotein were slightly higher in MMD patients, but within the range of normal values. To study the miRNA mechanism in MMD, we determined the serum miRNA expression profile through microarray analysis. First, we assessed miRNA expression profiles in the two pooled serum samples, and 94 miRNAs were differentially expressed (Table 3). Of these, 50 miRNAs were upregulated by more than 2 folds in MMD group compared with the control group, while 44 miRNAs were downregulated, also by more than 2 folds. Several miRNA families and clusters were detected. Let-7 family (let-7a, let-7b, let-7c, let-7g and let-7i), miR-17 family (miR-19a, miR-19b, miR-20a, miR-106b, miR-25 and miR-92a), and miR-15/16 cluster (miR-15a and miR-16) were elevated in serum of MMD patients. Let-7 family, miR-17 family and miR-15/16 cluster have been reportedly associated with tumor development [41], [42], [43]. The miRNA families and the cluster associated with MMD warrant further investigations.

**Table 2:** Characteristics of the study population.

Variable	Case	Control	P
Age(years)	34.40±10.29	29.50±3.38	0.192
Sex(Femal/Male)	5/5	4/6	0.653
WBC(×10∧9/L)	7.73±2.14	7.01±1.83	0.422
RBC(×10∧12/L)	4.22±0.44	4.50±0.74	0.329
GLU(mmol/L)	5.25±0.65	4.91±0.85	0.332
K^+^(mmol/L)	4.06±0.19	4.41±0.83	0.212
Na^+^(mmol/L)	142.00±2.91	141.3±3.96	0.657
ALT(U/L)	28.1±30.11	15.42±12.47	0.233
AST(U/L)	13.25±18.56	17.61±9.68	0.446
TB(umol/L)	10.25±3.75	6.10±3.47	0.019
DB(umol/L)	3.80±1.67	2.92±1.37	0.205
TC(mmol/L)	4.27±0.36	3.20±0.78	0.001
TG(mmol/L)	0.82±0.31	0.96±0.07	0.087
HDL(mmol/L)	1.05±0.21	1.03±0.09	0.797
LDL(mmol/L)	2.54±0.34	2.08±0.55	0.038

WBC: white blood cell; RBC: red blood cell; GLU: glucose; ALT: alanine aminotransferase; AST: aspartic acid amino transferase; TB: total bilirubin; DB: direct bilirubin; TC: total cholesterol; TG: triglyceride; HDL: high density lipoprotein; LDL: low density lipoprotein.

**Table 3:** Differentially expressed miRNAs in MMD.

Upregulated miRNAs				Downregulated miRNAs			
MiRNA	Fold	MiRNA	Fold	MiRNA	Fold	MiRNA	Fold
miR-106b	247.8	miR-151-3p	32.3	miR-3648	−207	miR-1305	−24.7
miR-140-3p	145.7	miR-361-5p	32.3	miR-125a-3p	−133	miR-595	−19.0
miR-320d	141.3	miR-1274b	29.6	miR-4299	−124	miR-30c-1*	−16.7
miR-29c	138.1	miR-146a	28.7	miR-1224-5p	−97.8	miR-769-3p	−15.2
miR-126	138	miR-103	28.5	miR-3692*	−81.6	miR-371-5p	−5.2
miR-142-3p	130.7	miR-101	28.5	miR-32*	−81.5	miR-4257	−3.9
let-7i	121.2	miR-29a	26.7	miR-3198	−76.9	miR-3195	−3.5
miR-320e	114.3	miR-145	26.6	miR-3156	−75.6	miR-765	−3.0
miR-122	101.1	miR-877*	19.4	miR-1469	−72.6	miR-3202	−2.8
miR-130a	98.51	miR-144	16.9	miR-1182	−71.9	miR-575	−2.8
miR-19a	97.4	miR-191*	13.3	miR-557	−67.6	miR-3679-5p	−2.8
miR-107	69.4	miR-19b	11.9	miR-1226*	−59.8	miR-1471	−2.4
miR-1290	67.0	miR-451	9.3	miR-601	−59.5	miR-4271	−2.4
miR-15b	61.1	miR-16	7.2	miR-3149	−59.3	miR-423-5p	−2.3
miR-30a	58.0	miR-320b	5.0	miR-4313	−57.8	miR-483-5p	−2.2
miR-1183	55.9	miR-25	4.6	miR-3945	−53.6	miR-3188	−2.2
miR-15a	46.6	miR-22	4.5	miR-202	−52.6	miR-574-5p	−2.1
miR-185	41.0	miR-21	3.5	miR-3180-5p	−51.1	miR-4298	−2.1
miR-338-3p	41.0	let-7b	3.4	miR-514b-5p	−48.3	miR-197	−2.0
let-7c	40.3	miR-720	3.2	miR-187*	−47.1		
miR-4286	39.1	miR-26a	2.9	miR-3605-5p	−42.4		
let-7g	37.9	miR-23a	2.7	miR-711	−35.1		
miR-483-3p	36.3	miR-92a	2.5	miR-2278	−30.9		
let-7a	34.1	miR-486-5p	2.5	miR-3652	−27.8		
miR-20a	33.8	miR-30e	2.2	miR-3646	−26.8		

**Figure 1:**
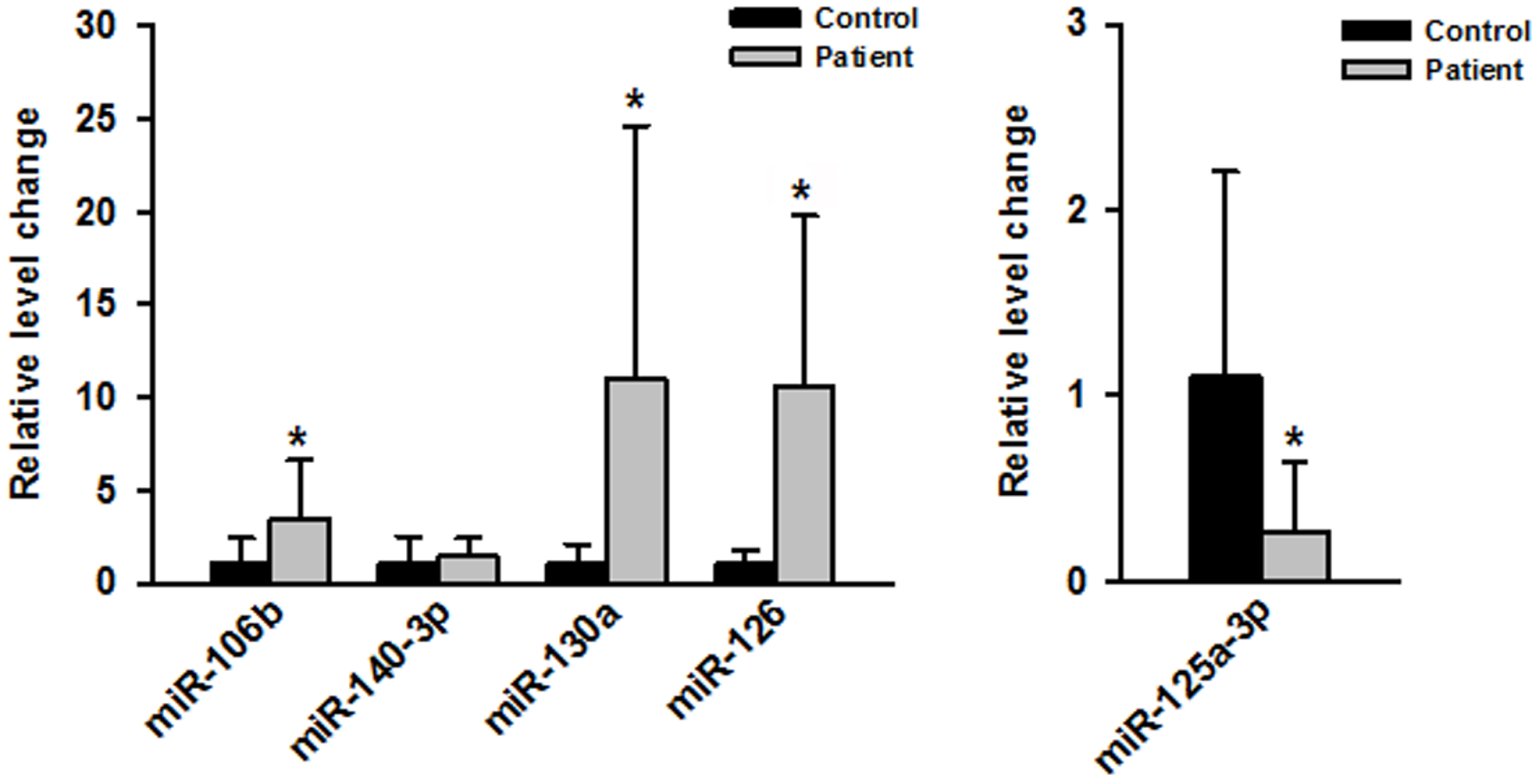
Real-time PCR of dysregulated miRNAs. Expression levels of miR-106b, miR-140-3p, miR-130a, miR-126 and miR-125a-3p in MMD serum were measured by real-time PCR and quantified as described in Methods. * P<0.05 compared with control group.

Serum miR-106b, miR-130a, miR-126 and miR-125a-3p were validated in independent MMD samples. Real-time PCR assay was used to further confirm the serum miRNA expression profile. The natural history of MMD may be associated with vascular endothelium and neovascularization [2], thereby justifying the focus of our experimental design. Using Pubmed search, we selected 5 associated miRNAs, including miR-106b [44], miR-140-3p [45], miR-130a [46], miR-126 [47] and miR-125a-3p [48], for further study. We then examined these miRNAs in serum from an independent cohort containing 10 MMD samples and 10 control samples. As shown in Fig 1, miR-106b, miR-130a and miR-126 were significantly upregulated (P<0.05), whereas miR-125a-3p was significantly downregulated in MMD serum (P<0.05). Our real-time PCR validation rate was relatively high (4/5).

### Potential functional targets

Since miRNAs negatively regulate the expression of their target genes, the next step was to screen the potential functional targets of the differentially expressed miRNAs. By searching and defining the target prediction program, we identified 1989 potential functional targets that may participate in MMD pathogenesis. GO analysis of biological process showed that potential functional targets were enriched in metabolic processes, transcription and signal transduction. The top 10 terms of biological processes were listed in Table 4.

**Table 4:** Top 10 biological processes as potential functional targets (1989).

Term	Count	P Value
protein amino acid phosphorylation	116	1.00E-08
phosphate metabolic process	150	1.97E-07
phosphorus metabolic process	150	1.97E-07
regulation of transcription	338	4.49E-07
phosphorylation	125	1.19E-06
enzyme linked receptor protein signaling pathway	65	1.25E-06
transcription	278	1.61E-06
cellular macromolecule catabolic process	112	7.95E-06
response to peptide hormone stimulus	35	1.18E-05
response to insulin stimulus	26	1.81E-05

### mTOR pathway might be regulated by miRNAs and involved in MMD pathogenesis

**Figure 2:**
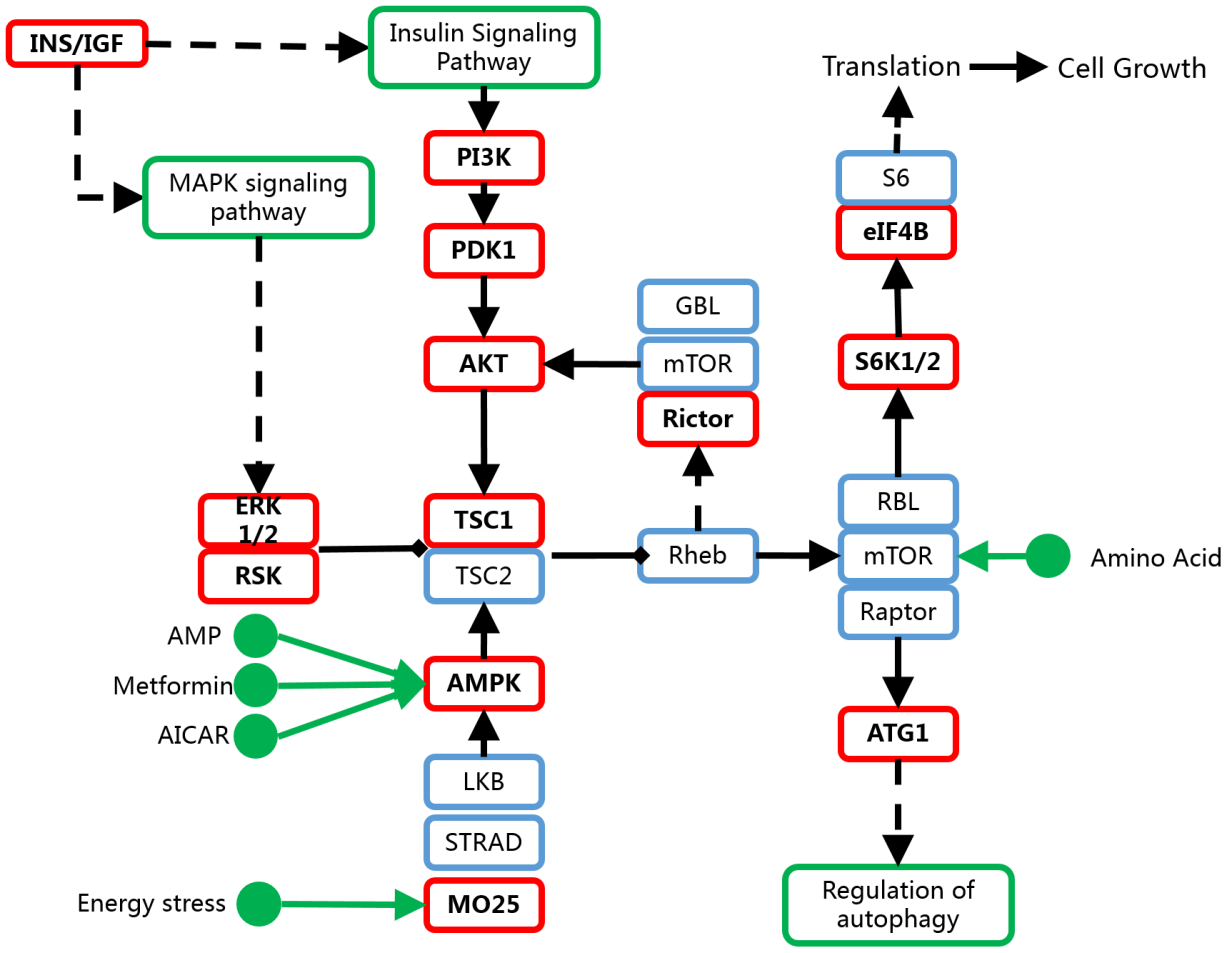
Targets of the differentially expressed miRNAs in mTOR signaling pathway. Red boxes indicate potential functional targets of the differentially expressed miRNAs in MMD (based on Kanehisa Laboratories on 7/25/12, from KEGG).

Pathway analysis revealed 18 different pathways corresponding to the target genes (P<0.05, Table 5). The most enriched pathway was mTOR signaling pathway (Fig 2) with 16 potential functional targets. Recent data suggest that various matrixins, cytokines and angiogenic factors in plasma are involved in the pathogenesis of MMD, including MMP-3, MMP-9, MCP-1, VEGF, PDGF-BB, etc [20], [ 49]. These factors may be regulated by MAPK signaling and VEGF signaling, which are associated with mTOR pathway.

**Table 5:** Enriched pathways for potential functional targets (1989).

Term	Count	P Value
mTOR signaling pathway	16	8.05E-05
Ubiquitin mediated proteolysis	28	2.47E-04
Adherens junction	19	3.14E-04
Endocytosis	33	7.26E-04
Focal adhesion	35	8.35E-04
T cell receptor signaling pathway	22	0.001419
Oocyte meiosis	21	0.004158
Aldosterone-regulated sodium reabsorption	11	0.004868
Axon guidance	23	0.006051
Progesterone-mediated oocyte maturation	17	0.007885
ErbB signaling pathway	17	0.008822
Gap junction	17	0.010962
Regulation of actin cytoskeleton	32	0.01563
Biosynthesis of unsaturated fatty acids	7	0.015886
TGF-beta signaling pathway	16	0.019483
Neurotrophin signaling pathway	20	0.029869
Long-term depression	13	0.032527
Insulin signaling pathway	21	0.036219

### RNF213 and BRCC3- associated miRNAs

**Figure 3:**
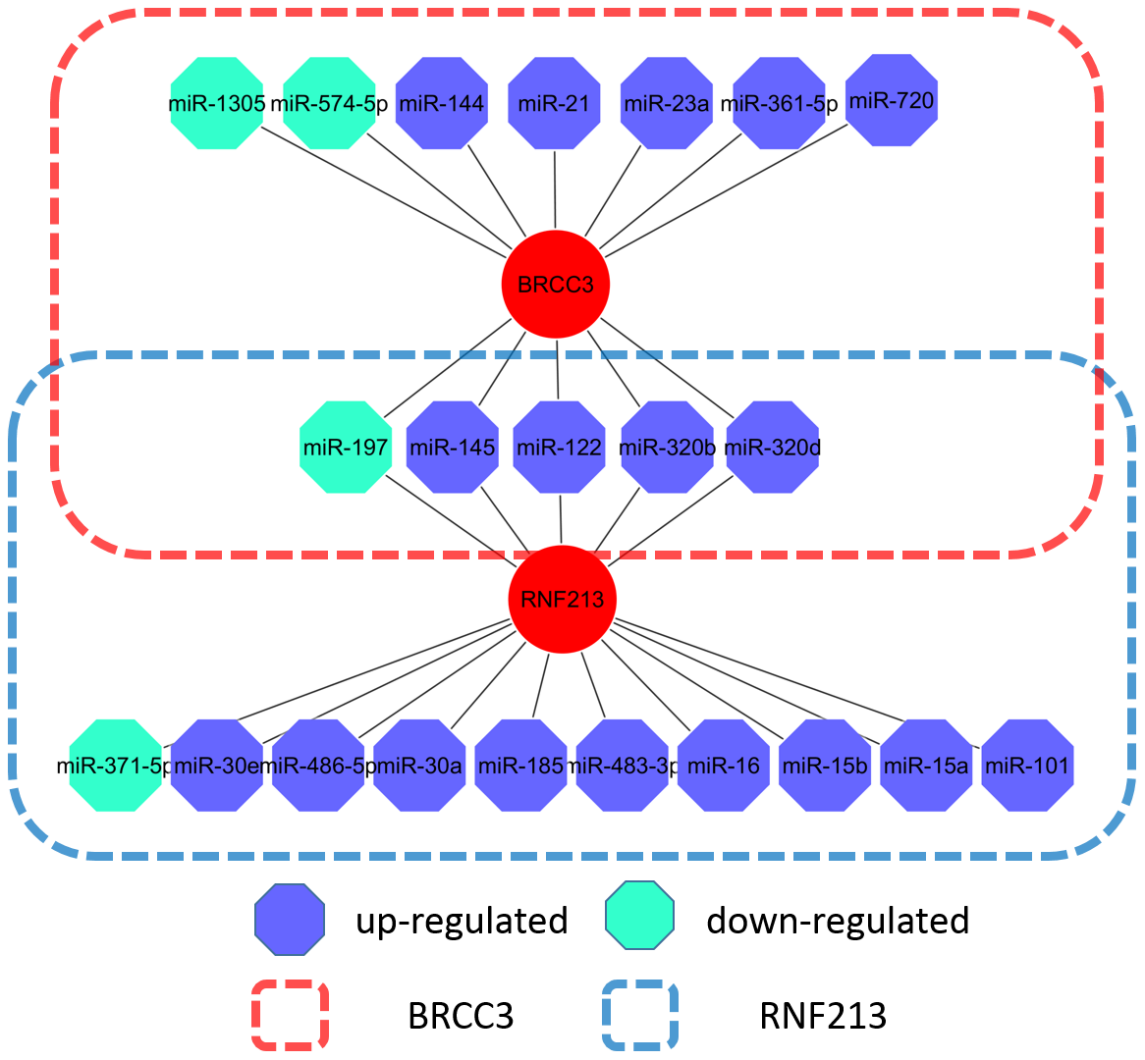
Network of RNF213- and BRCC3-associated miRNAs in MMD.

To explore unknown angiogenesis-related miRNAs, we performed a Pubmed search for known genes. Recent data have shown that RNF213 is a susceptible gene for MMD. Knockdown of RNF213 in zebrafish causes irregular wall formation in trunk arteries, with abnormal sprouting vessels [50], [ 51], [ 52]. We found that RNF213 was the potential target of 16 differentially expressed serum miRNAs in MMD, involving upregulation of 14/16 (87.5%) serum miRNAs (Fig 3). The 14 over-expressed miRNAs may synergistically interact with the 3′UTR of RNF213 to inhibit RNF213 protein expression, thereby modulating MMD pathogenesis.

Another study has reported that syndromic moyamoya was associated with Xq28 deletions involving BRCC3, leading to defective angiogenesis in zebrafish [53]. Of the 94 aberrant miRNAs, 13 miRNAs potentially target BRCC3, including 10 upregulated and 3 downregulated miRNAs (Fig 3). The 13 differentially expressed serum miRNAs inhibit BRCC3 protein expression at the posttranscriptional level, resulting in abnormal angiogenesis in MMD.

## Discussion

Extensive data indicate that serum miRNAs act as potential biomarkers for various diseases [54]. However, until now, no study has reported detail serum miRNAs in MMD. Neurosci Lett recently published a paper which demonstrated a higher frequency of the CT+CC genotype of the SNP rs11614913 in miR-196a2C>T [52], suggesting that miR-196a2 may play an important role in the pathogenesis of Moyamoya disease. It's worth noting that although annexin A1 (lipocortin1, ANXA1), a main targeted gene of miR-196a, was identified to be linked with increased multiple malignant tumors in brain models of ischemia and reperfusion injury, the SNP rs11614913 in miR-196a2C>T would not affect the function inhibiting targeted genes expression, because it was not present in the seed-sequences of miR-196a. Actually, the data of plasma miRNA microarray showed no differences in miR-196a between the two groups. However, whether SNP rs11614913 in miR-196a2C>T could be present in the Moyamoya patients involved in our investigation should also be confirmed in the future. A genome-wide array was used in our study to screen 10 patients with MMD for serum miRNAs. For the first time, we detected a serum miRNA signature for MMD and found that mTOR pathway and RNF213 and BRCC3-associated miRNAs play an important role in MMD.

In our study, we confirmed that serum miR-106b, miR-130a, miR-126 and miR-125a-3p were dysregulated in MMD. Human circulating miR-126 has been reported as a potential, novel indicator for acute myocardial infarction (AMI) [55]. Decreased miR-126 in plasma from symptomatic AMI patients compared with healthy subjects correlated with expression of cardiac troponin I (cTnI). Further, in hepatocellular carcinoma, upregulation of miR-125a significantly repressed the expression of vascular endothelial growth factor A (VEGF-A) and matrix metalloproteinase 11 (MMP11), both in vitro and in vivo [56]. The mechanism of aberrant serum miRNAs involved in MMD warrants further investigations.

The mechanism and function of serum miRNAs remain unclear. Recently, miRNAs have been reportedly transported in microvesicles and involved in gene silencing [57]. According to Skog et al., glioblastoma-derived RNA contained in microvesicles was functional and transported by human brain microvascular endothelial cells (HBMVEC) in cultures [28], [ 58]. If this hypothesis holds true, differentially expressed serum miRNAs in MMD may be delivered by HBMVEC to regulate MMD associated genes. Loss of RNF213 and BRCC3 has been associated with MMD pathogenesis [50], [ 53], [ 57]. Here, we showed that most RNF213- and BRCC3-associated miRNAs were elevated in serum of MMD patients, repressed RNF213 and BRCC3 protein expression in HBMVEC, and modulated HBMVEC angiogenesis. Therefore, searching for known angiogenesis-associated genes might be a better way to identify potentially unknown angiogenesis-associated miRNAs.

In conclusion, our study revealed, for the first time, that serum miRNAs were differentially expressed in MMD. The miR-106b, miR-130a, miR-126 and miR-125a-3p play a critical role in the pathogenesis of MMD. The RNF213- and BRCC3-associated miRNAs may significantly contribute to the development of MMD.

However, the study limitations are related to the target mRNA(s) of the differentially expressed miRNAs. Experimental validation is required to confirm the targets of the differentially expressed miRNAs predicted by computational algorithms. Another limitation involves the relatively small cohort size. A similar study with larger cohorts of MMD and corresponding control sample may improve the reliability of our findings.
